# Geographic Variation in the Chemical Composition and Antioxidant Properties of Phenolic Compounds from *Cyclocarya paliurus* (Batal) Iljinskaja Leaves

**DOI:** 10.3390/molecules23102440

**Published:** 2018-09-24

**Authors:** Yang Liu, Pei Chen, Mingming Zhou, Tongli Wang, Shengzuo Fang, Xulan Shang, Xiangxiang Fu

**Affiliations:** 1 College of Forestry, Nanjing Forestry University, Nanjing 210037, China; lyang_188@sina.com (Y.L.); chen951201@sina.com (P.C.); zhoumingming222@sina.com (M.Z.); shangxulan@njfu.edu.cn (X.S.); xxfu@njfu.edu.cn (X.F.); 2 Department of Forest and Conservation Sciences, University of British Columbia, 3041- 2424 Main Mall, Vancouver, BC V6T 1Z4, Canada; tongli.wang@ubc.ca; 3 Co-Innovation Center for Sustainable Forestry in Southern China, Nanjing Forestry University, Nanjing 210037, China

**Keywords:** *Cyclocarya paliurus*, antioxidant, phenolics, geographic origin, HPLC

## Abstract

*Cyclocarya paliurus* has been widely used as an ingredient in functional foods in China. However, the antioxidant properties of phenolic compounds and the effect of the plant origin remain unclear. The present study evaluated the geographical variation of this plant in term of its phenolic composition and antioxidant activities based on leaf materials collected from five regions. high-performance liquid chromatography (HPLC) analysis showed that there are three major components, quercetin-3-*O*-glucuronide, kaempferol-3-*O*-glucuronide, and kaempferol-3-*O*-rhamnoside, and their contents varied significantly among sampling locations. The investigated phenolic compounds showed substantial antioxidant activities, both in vitro and in vivo, with the highest capacity observed from Wufeng and Jinzhongshan. Correlation analysis revealed that quercetin and kaempferol glycosides might be responsible for the antioxidant activities. Our results indicate the importance of geographic origin, with sunny hours and temperature as the main drivers affecting the accumulation of *C. paliurus* phenolics and their antioxidant properties.

## 1. Introduction

It is well known that excess reactive oxygen species (ROS) can lead to so-called oxidative stress in the human body, which is a major cause of aging and disease [1]. Treatments with synthetic antioxidants often have toxic or side effects [2]. Hence, attention in recent years has focused on screening natural antioxidants from plants, as they can help maintain the biological balance between oxidation and antioxidation [1]. Polyphenols, a large category of secondary plant metabolites, have been widely studied for their biological functions in combating chronic disease due to their high antioxidant activities [3,4,5]. In this context, fruits, vegetables, and plants that are rich in polyphenols such as *Ginkgo biloba*, *Crataegus pinnatifida*, and *Cyclocarya paliurus*, are receiving increasing attention from pharmacological researchers and nutritionists [6,7,8].

*Cyclocarya paliurus* (Batal) Iljinskaja is a multifunction tree species that belongs to the Juglandaceae family and is mainly distributed in the subtropical highlands of China [9]. Leaves of this plant have been used in China as functional foods or a nutraceutical tea for the treatment of hyperhidrosis, hypertension, and diabetes mellitus [8,10], and have been approved as a new food raw material by the National Health and Family Planning Commission of China in 2013. Studies have also revealed that the leaves of *C. paliurus* contain abundant bioactive compounds, including triterpenoids, polysaccharides, and phenolic compounds [11,12]. Thus, many studies were focused on the identification of these substances of this plant and their pharmacological effects [4,13]. For example, three types of flavonoids, isoquercitrin, kaempferol, and quercetin, as well as a few selected triterpenoids, have been previously identified [8,12,13].

Diabetes mellitus (DM) is a severe worldwide chronic disease that leads to complications such as renal disease, nontraumatic limb amputation, and blindness [14,15]. Previous studies have revealed that the onset of diabetes in both experimental animals and clinical patients was closely associated with oxidative stress [16,17]. It was reported that ROS could lead to the destruction of many tissues, which then contributes to the induction of diabetes or its complications [18]. Thus, antioxidant enzymes, such as superoxide dismutase (SOD) and glutathione peroxidase (GSH-px) are often assayed in vivo to evaluate the antioxidant ability of plant extracts as they can provide essential assistance in scavenging free radicals [19,20].

It is generally accepted that the antioxidant activity of phenolic compounds is often related to the chemical composition of individual compounds, which is dependent on a variety of factors, such as geographic variation [21], harvest time [11], environmental and agronomic conditions [22], the botanical parts of plants [23], and extraction methods [10]. Recent studies have reported that genotypes, light conditions, and fertilization levels significantly affect flavonoid accumulation in *C. paliurus* [12,22]. It was also demonstrated that extract parameters such as temperature, the material to solvent ratio, extraction time, and solvents have a significant impact on the yield and the antioxidant activity of *C. paliurus* flavonoids [10]. However, to our best knowledge, antioxidant activities and the chemical composition of *C. paliurus* phenolic compounds have not been previously studied. Furthermore, no knowledge of the geographic variation in leaf phenolics is available for the natural populations of this species. 

The objective of this study was to determine the yield and composition of phenolic compounds in *C. paliurus* leaves collected from five geographical locations in China, and to evaluate their antioxidant capacity. Our evaluations were conducted in both in vitro and in vivo systems (2,2-diphenyl-1-picrylhydrazyl (DPPH) radical-scavenging ability, ferric-reducing antioxidant power (FRAP) assay, and SOD and GSH-px activities in a diabetic mouse model). The correlation between the obtained antioxidant activities and the studied phenolic contents was also evaluated. We hypothesized that significant geographic variations exist in the phenolic compositions and antioxidant activities of *C. paliurus* extracts, but the magnitude of antioxidant activities mainly depend on some key phenolic components in the extracts. The results of this study offer opportunities to identify *C. paliurus* populations with targeted health-promoting compounds and to establish future plantations with better pharmaceutical and food uses.

## 2. Results and Discussion

### 2.1. Phytochemical Composition

**Figure 1:**
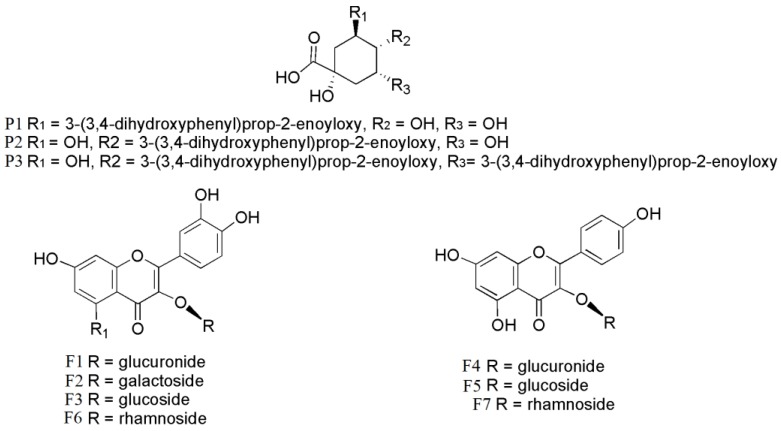
Chemical structures of the 10 quantitative compounds in leaves of *C. paliurus*: (1) 3-*O*-caffeoylquinic acid; (2) 4-*O*-caffeoylquinic acid; (3) quercetin-3-*O*-glucuronide; (4) quercetin-3-*O*-galactoside; (5) isoquercitrin; (6) kaempferol-3-*O*-glucuronide; (7) kaempferol 3-*O*-glucoside; (8) quercetin-3-*O*-rhamnoside; (9) 4,5-di-*O*-caffeoylquinic acid; (10) kaempferol-3-*O*-rhamnoside.

In order to identify the phenolic compounds responsible for the antioxidant activities, the phytochemical composition of *C. paliurus* extracts was clarified by using both quantitative and qualitative analyses through high-performance liquid chromatography coupled with quadrupole time-of-flight mass spectrometry (HPLC-Q-TOF-MS). The data of retention time, and MS and MS^2^ fragment ions are summarized in Table 1. The chemical structures of the 10 components are shown in Figure 1. We found that extracts from different locations were rich in flavonoids (Table 2), which yielded from 2.30 mg/g to 6.88 mg/g. The phenolic yields among the five locations studied were significantly different. The highest content of total phenolics (7.89 ± 0.02 mg/g dry weight (DW)) was found in the extract from WF, while the highest content of total flavonoids (6.87 ± 0.01 mg/g, DW) was observed in the extract from JZS. The five locations were ranked in the following order: WF > JZS > MW > MC > SN. 

**Figure 2:**
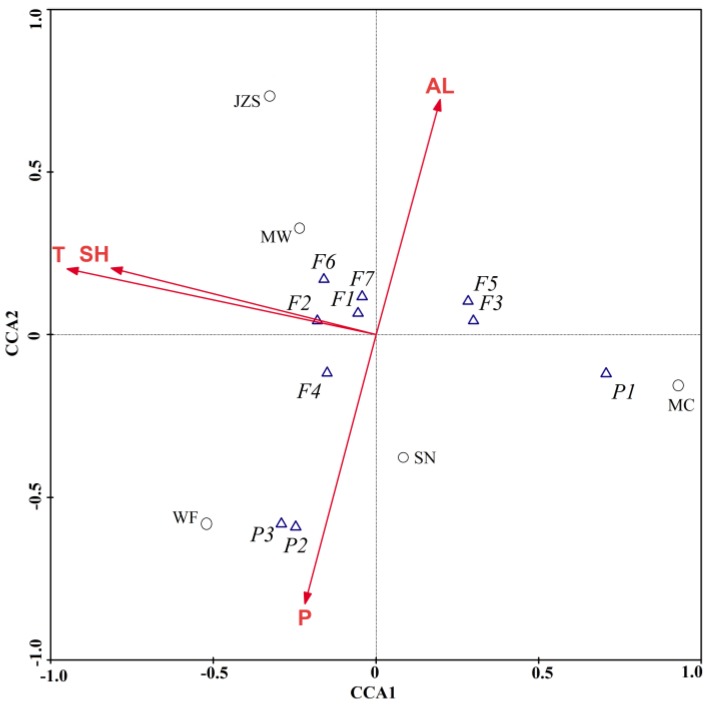
Canonical correspondence analysis biplot of *C. paliurus* phenolics, linking contents of the individual components, geographical locations (JZS, MC, WF, MW, and SN) and their bioclimatic indices. T: mean annual temperature; SH: mean annual sunshine hours; AL: altitude; P: mean annual precipitation; P1: 3-*O*-caffeoylquinic acid; P2: 4-*O*-caffeoylquinic acid; P3: 4,5-di-*O*-caffeoylquinic acid; F1: quercetin-3-*O*-glucuronide; F2: quercetin-3-*O*-galactoside; F3: isoquercitrin; F4: kaempferol-3-*O*-glucuronide; F5: kaempferol-3-*O*-glucoside; F6: quercetin-3-*O*-rhamnoside; F7: kaempferol-3-*O*-rhamnoside.

The quantities of individual phytochemicals were also given in Table 2. Three phenolic acids and seven flavonoids were identified. Meanwhile, quercetin-3-*O*-glucuronide (0.78–2.38 mg/g DW), kaempferol-3-*O*-glucuronide (0.51–1.88 mg/g DW), and kaempferol-3-*O*-rhamnoside (0.68–2.30 mg/g DW) were found to be the predominant components. These results were comparable to those of a previous study [13]. Furthermore, the canonical correspondence analysis (CCA) test in our study showed that the higher yields of phenolic compounds from JZS, WF, and MW were associated with increasing mean annual temperatures and sunshine hours (Figure 2). A previous study also indicated that genetic diversity was low among these *C. paliurus* populations based on the simple-sequence repeat (SSR) and inter-SSR (ISSR) tests [24]. Thus, compared with environmental factors, the genotype may have a slight impact on phytochemical accumulation, which is inconsistent with the results of Sosa et al. [25]. Overall, higher radiance and drier climate in these locations might lead to increased use of secondary metabolites, such as phenolic acids and flavonoids, by plants to protect cells from oxidative damages caused by ROS [22].

### 2.2. Variation in Antioxidant Activity

**Figure 3:**
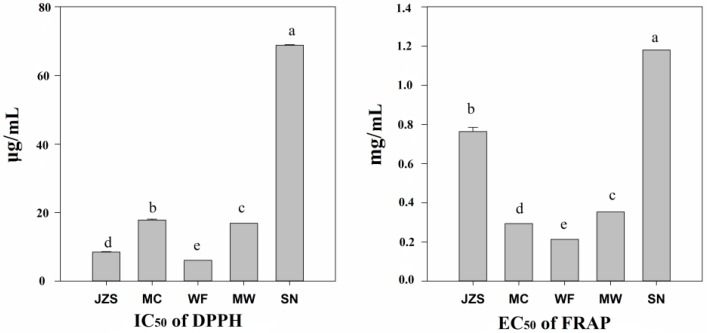
Antioxidant activities of *C. paliurus* phenolics from different locations (JZS, MC, WF, MW, and SN) in vitro, using two different testing systems. Different letters indicate significant differences (*p* < 0.05) between locations.

The DPPH and FRAP methods are commonly applied to determine the antioxidant activities of phenolic compounds by measuring the capacity of plant extracts to donate hydrogen of DPPH or reduce ferric iron to ferrous in vitro [26]. The antioxidant activity from DPPH and FRAP tests is presented in Figure 3. The investigated *C. paliurus* phenolic compounds showed substantial antioxidant activities, although the two tests yielded different values of antioxidant activities. IC_50_ values of DPPH and EC_50_ values of FRAP ranged from 6.1 to 68.8 μg/mL, and from 0.29 to 1.18 mg/mL, respectively. The highest antioxidant of DPPH and FRAP tests was observed from the WF location, while the lowest antioxidant activity was observed from the SN region. Compared with other species, the antioxidant activity of *C. paliurus* phenolics is much higher than that of *Thymus daenensis* (IC_50_ of DPPH: 273.4 μg/mL), *Folium eucommiae* (IC_50_ of DPPH: 100.0 μg/mL), *Copaifera langsdorffii* (IC_50_ of DPPH: 510.0 μg/mL), *Ginkgo biloba* (IC_50_ of DPPH: 40.7 μg/mL), and comparable with that of *Fagopyrum tataricum* (IC_50_ of DPPH: 8.4 μg/mL), *Anadenanthera peregrina* (IC_50_ of DPPH: 11.6 μg/mL), and *Algerian Mentha* (IC_50_ of DPPH: 16.2 μg/mL) [6,23,27,28,29,30].

**Figure 4:**
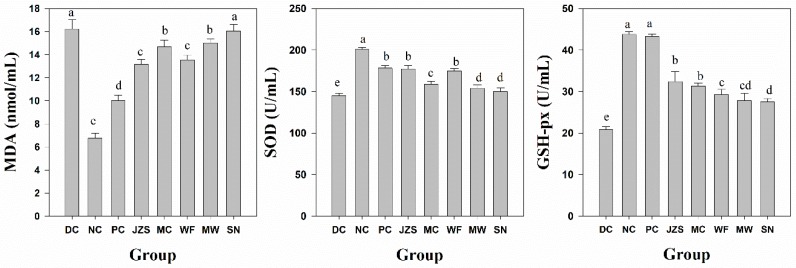
Antioxidant index of *C. paliurus* phenolics from sampling locations (JZS, MC, WF, MW, and SN) in diabetic model mice. Columns having different letters are significantly different at *p* < 0.05. DC: diabetic control group; NC: normal control group; PC: positive control group.

Malonaldehyde (MDA) is an important index that represents the degree of lipid peroxidation in tissue [31]. Compared to normal mice, MDA levels in STZ-induced diabetic mice were significantly increased (Figure 4), which was in consistence with a previous report [29]. Treatment with *C. paliurus* phenolics from JZS, MC, WF, and MW significantly decreased MDA levels, which revealed their protective effects in diabetes treatment by reducing oxidative stress (Figure 4). It is well known that the activities of antioxidant enzymes, such as SOD and GSH-px, are very low in diabetic animals as compared to those of normal animals [32]. Our study showed that treatment with *C. paliurus* extracts markedly restored SOD and GSH-px activities in diabetic mice. Results also revealed that variation of the antioxidant activities of *C. paliurus* phenolics from different locations in vivo was significant (Figure 4), with the best effect observed from the JZS treated group. 

**Figure 5:**
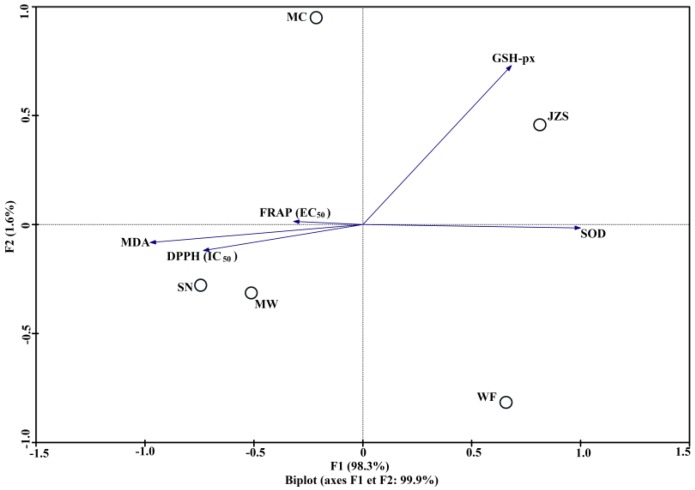
Principal component analysis for studied locations (JZS, MC, WF, MW, and SN) performed on values of both in vitro and in vivo antioxidant indices (Biplot according to the first two axes).

To further analyze variability of antioxidant activities among *C. paliurus* phenolics, principal component analysis (PCA) was carried out based on matrix-linking the values of the antioxidant index to different locations. Results showed that the first two axes explained 99.9% of the total variability, with the most variability explained by the horizontal axis (98.3%) (Figure 5). PCA results indicated that the studied antioxidant index displayed different correlations with the first two axes. Enzyme activities of SOD (loading, 0.840) and GSH-px (loading, 0.732) were correlated to the horizontal axis, which achieved significantly higher values than that of MDA (loading, 0.201), IC_50_ of DPPH (loading, 0.294), and EC_50_ of FRAP (loading, 0.400). The PCA biplot also showed that extracts from JZS and WF characterized by higher contents of phenolic and flavonoid compounds showed the highest antioxidant activities, both in vitro and in vivo. This variability of antioxidant properties offers an opportunity to collect *C. paliurus* leaves from these populations for better pharmaceutical and food uses in the future.

### 2.3. Correlation of Phenolic Compounds and Antioxidant Activity

Correlation between the obtained antioxidant activities and studied phenolic contents is shown in Table 3. Positive correlations were presented between the contents of total phenolic or total flavonoid compounds and antioxidant ability. Total phenolics were negatively correlated with IC_50_ of DPPH (*r* = −0.991, *p* < 0.01) and MDA (*r* = −0.878, *p* < 0.05). Similarly, total flavonoids were also negatively correlated with IC_50_ of DPPH (*r* = −0.950, *p* < 0.05) and MDA (*r* = −0.903, *p* < 0.05). These negative relationships revealed the antioxidant effects of total phenolics and total flavonoids of this plant, which can be used as an alternative additive in foods and pharmaceuticals [4]. 

According to the presented analysis, quercetin-3-*O*-glucuronide (F1) and quercetin-3-*O*-rhamnoside (F6) seemed to have a higher contribution to the antioxidant activities of *C. paliurus* in in vitro assays (Table 3). Additionally, strong correlation was observed between the antioxidant tests in vivo (SOD, GSH-px, and MDA values) and the contents of kaempferol-3-*O*-glucoside (F5) and kaempferol-3-*O*-rhamnoside (F7). These results suggest that the antioxidant effects of *C. paliurus* might be mainly dependent on these individual phenolics. Previous studies have also indicated that quercetin or kaempferol glycosides from plants exhibited various activities such as antitumor, antioxidant, immunoregulatory, and antihyperglycemic effects [4,33,34,35]. However, in order to determine the individual compounds responsible for antioxidant activities, isolation and purification should in the future be done using chromatographic techniques.

## 3. Materials and Methods

### 3.1. Plant Material

Five geographical locations were selected for *C. paliurus* sampling based on its major distribution (ranging from 23° N to 35° N for latitude, and 100° E to 122° E for longitude) in China. Voucher specimens were deposited in Silviculture Lab of Nanjing Forestry University (Voucher code: 2011GX, 2011HB, 2011SC, 2011HN, 2011ZJ). Detailed climatic and geographic information of these locations (Jinzhongshan, JZS; Muchuan, MC; Wufeng, WF; Meiwu, MW; and Suining, SN) is shown in the Table 4. Information on sample collecting and pretreatment was the same as our previous study [36]. Briefly, leaf sampling was carried out in September 2014. At each location, 6–30 sample trees (dominant or codominant trees) were chosen based on stem form, tree age, and population size. Leaves (about 400 g per tree from the middle crown) of each location were mixed and dried to constant weight in the lab, and then ground into fine powder for the preparation of *C. paliurus* extracts.

### 3.2. Preparation of C. paliurus Extracts

Extraction of *C. paliurus* samples was carried out as described previously, with slight modifications, in August 2015 [4]. The leaf powder (about 75 g) from each sample was extracted with 80% ethanol (1000 mL) and incubated at 90 °C for 1 hour. Afterward, the mixture was shaken at 25 °C for 15 min. Centrifugation for the mixture was done at 8000 g for 10 min, and the supernatants were then evaporated at 40 °C to yield an 80% ethanol extract (JZS: 36.5 g, 48.7% yield; MC: 37.6 g, 50.1% yield; WF: 36.8 g, 49.1% yield; MW: 36.1 g, 48.1% yield; SN: 36.4 g, 48.5% yield). All extracts were stored in the lab at 4 °C before antioxidant tests and chemical analysis.

### 3.3. HPLC-Q-TOF-MS Confirmation

Liquid chromatography (LC)-mass spectrometry (MS) analysis was carried out to confirm the peak identities. The identification was performed on an Agilent 6520 Q-TOF mass spectrometer system equipped with a diode array detector (DAD) and electrospray interface (ESI) (Agilent Technologies, Santa Clara, CA, USA). The MS system was operated in negative ionization modes with the mass scan range set at *m*/*z* 100-1200. The mass spectral parameters were a gas temperature of 300 °C; gas flow of 10 L/min; nebulizer pressure of 30 psi; capillary voltage of 4000 V; cone voltage of 100 V; and collision voltage: 60 V. The chromatographic conditions were same as those described above. Agilent Mass Hunter version B.04.00 software was used for data acquisition and processing. Peaks were identified on the basis of comparison of retention times and MS spectra with standards.

### 3.4. Determination of Phenolic Compounds by HPLC

Phenolic profiles of *C. paliurus* from different locations were measured using an HPLC system according to a previous method, with slight modifications, in September 2015 (Waters, Milford, MA, USA) [13]. Separation for *C. paliurus* phenolics was carried out on an X-Bridge C18 column by a stepwise elution with acetonitrile containing 0.01% formic acid (solution A) and water containing 0.01% formic acid (solution B). The gradient elution included 0–13 min, 8% A; 13–28 min, 19% A; and 28–40 min, 21% A. The flow rate was kept at 1.0 mL/min and the injection volume was 10 μL. Meanwhile, column temperature was kept at 45 °C and the wavelength for detection was 205 nm. Contents of individual compounds were quantified from their external standards. The representative HPLC chromatogram of 80% ethanol extract of the *C. paliurus* leaves collected from (a) JZS, and (b) mixed standards can be seen in the supplementary file (Figure S1). Contents of total phenolic acids (TPA) and total flavonoids (TF) of the samples were the sums of their individuals, while total phenolic contents (TPC) were the sum of all individual phenolics detected. The reference standards of 3-*O*-caffeoylquinic acid, 4-*O*-caffeoylquinic acid, isoquercitrin, kaempferol-3-*O*-glucuronide, 4,5-di-*O*-caffeoylquinic acid, quercetin-3-*O*-glucuronide, quercetin-3-*O*-rhamnoside, quercetin-3-*O*-galactoside and kaempferol-3-*O*-glucoside (purity > 98%) were purchased from BioBioPha Co., Ltd. (Kunming, China), and kaempferol-3-*O*-rhamnoside (purity > 98%) was isolated and purified from the laboratory of China Pharmaceutical University (Nanjing, China) [13].

### 3.5. Antioxidant Activity In Vitro

#### 3.5.1. DPPH Radical-Scavenging Activity

The DPPH radical-scavenging activities of *C. paliurus* phenolics were carried out as described by a previous study, with some modifications, in September 2015 [37]. *C. paliurus* extracts were dissolved in methanol. Samples of various concentrations (0.1–2.0 mg/mL, 2 mL) were added to the DPPH methanol solution (0.5 mM, 2 mL). Afterward, incubation of the mixture was done at 25 °C for 30 min in a water bath. Sample absorbance was then measured at 517 nm against a blank. The radical-scavenging activities of *C. paliurus* phenolics were calculated from the equation: radical scavenging activity (%) = [(A_0_ − A_1_)/A_0_] × 100, where A_0_ was the absorbance of the DPPH solution without the sample and A_1_ was the absorbance of the tested samples. The assay was carried out in triplicate. The extract concentration at 50% radical-scavenging activity (IC_50_) was then calculated from the graph of scavenging activities against six gradient concentrations.

#### 3.5.2. FRAP

The reducing power of *C. paliurus* phenolics was determined according to the Fe^3+^ reduction method with modifications in September 2015 [38]. Different ethanolic concentrations (1 mL, 0.2–2.0 mg/mL) of the *C. paliurus* extracts were mixed with 2 mL phosphate buffer (0.2 M, pH 6.6) and 2 mL potassium ferricyanide (K_3_Fe(CN)_6_, 1%). Afterward, incubation of the mixture was done at 50 °C for 20 min in a water bath. Trichloroacetic acid (4 mL, 10%) was then added and the mixture was centrifuged at 8000 rpm for 5 min. Finally, the supernatants of the solution (2 mL) were mixed with distilled water (2 mL) and ferric chloride (FeCl_3_; 0.4 mL, 1%), and sample absorbance was measured at 700 nm. The extract concentration at 0.5 absorbance (EC_50_) was calculated by plotting absorbance at 700 nm against the six tested gradient concentrations.

### 3.6. Antioxidant Activity In Vivo

#### 3.6.1. Animals and Experimental Design

C57BL/6 diabetic mice (male, 17.9–18.1 g, certificate number: SCXK (HU) 2013-0018) were used for the in vivo antioxidant analysis of *C. paliurus* phenolics at the same time. Animal resources, management, and the development of diabetes were the same as our previous study, following the guidelines and policy set forth by the Chinese Experimental Animals Administration Legislation (Ethical Protocol #SL-008-01) [4]. Diabetic mice were then treated with *C. paliurus* extract from each of the five locations (JZS, MC, WF, MW, and SN), respectively (8 g/kg body weight once a day, *n* = 6). Diabetic mice treated with distilled water (10 mL/kg body weight) or Metformin hydrochloride tablets (250 mg/kg body weight) were defined as negative (DC) and positive (PC) control groups (*n* = 6), respectively [39]. Meanwhile, normal mice treated with distilled water (10 mL//kg body weight) was defined as the normal control (NC) group (*n* = 6).

#### 3.6.2. Biochemical Assay

Animals in each group of 2.5.1 were administered by gavage with a basal control diet once a day for 28 days. Blood samples were then collected from the abdominal aortic at the end of the trial, and levels of SOD, GSH-px, and MDA content were then measured using commercial kits.

### 3.7. Data Analysis

Statistical analysis was performed using Duncan multiple range tests (SPSS, Chicago, IL, USA, *p* < 0.05). The impact of geographical and environmental factors on phenolic variability was measured using CCA (multivariate statistical package (MVSP) V5.2). To assess the variability of antioxidant activities of phenolics among sampling locations, we used PCA based on matrix-linking values of the antioxidant index, both in vitro and in vivo, to different locations.

## 4. Conclusions

Our results confirmed the hypothesis that geographical location significantly affects the phenolic composition and antioxidant activities of *C. paliurus* extracts, and the magnitude of antioxidant activities mainly depends on some key phenolic components in the extracts. The highest antioxidant activity in vitro was observed from the Wufeng location, and the lowest activity was observed from the Suining region. Results also demonstrated that variation in the antioxidant activities of *C. paliurus* phenolics in vivo was significant among different locations, and the best effect was observed from the JZS location. Correlation analysis revealed that quercetin-3-*O*-glucuronide and quercetin-3-*O*-rhamnoside, and kaempferol-3-*O*-glucoside and kaempferol-3-*O*-rhamnoside might be responsible for the antioxidant capacity both in vitro and in vivo, respectively. These results suggested the importance of growing environment selection for better use of this plant in pharmaceutical and food industries. To determine the specific compounds responsible for antioxidant activities, isolation, purification, and bioactivity analysis of *C. paliurus* phenolics should be carried out in the future.

## Figures and Tables

**Table 1:** The data of retention time, and MS and MS^2^ fragment ions of 10 phenolics from leaves of *C. paliurus* by developed high-performance liquid chromatography coupled with quadrupole time-of-flight mass spectrometry (HPLC–Q–TOF–MS).

Compounds	t_R_ (min)	[M − H]^−^	MS/MS Fragment Ion (*m*/*z*)	Formula
3-*O*-caffeoylquinic acid (P1)	7.4	353.9874	191.0554	C_16_H_8_O_9_
4-*O*-caffeoylquinic acid (P2)	7.8	353.0875	191.0552; 179.0339; 135.0444	C_16_H_8_O_9_
quercetin-3-*O*-glucuronide (F1)	14.9	477.0673	301.0351; 178.9979; 151.003	C_21_H_18_O_13_
quercetin-3-*O*-galactoside (F2)	15.4	463.0888	301.0343; 271.0244; 178.9979; 151.0029	C_21_H_20_O_12_
isoquercitrin (F3)	15.8	463.0888	301.0338; 271.0239; 178.9981; 151.0029	C_21_H_20_O_12_
kaempferol-3-*O*-glucuronide (F4)	17.6	461.9727	286.0431; 285.0401; 113.0235; 85.0296	C_21_H_18_O_12_
kaempferol-3-*O*-glucoside (F5)	18.5	447.0935	285.0388; 284.0319; 255.0295	C_21_H_20_O_11_
quercetin-3-*O*-rhamnoside (F6)	18.8	447.0973	301.0337; 285.0388; 255.0294; 217.0133	C_21_H_20_O_11_
4,5-di-*O*-caffeoylquinic acid (P3)	19.8	515.1199	353.0864; 191.0553; 179.0342; 135.0443	C_25_H_24_O_12_
kaempferol-3-*O*-rhamnoside (F7)	23.4	431.0981	285.0394; 255.0295; 227.0342	C_21_H_20_O_10_

**Table 2:** Phenolic contents in *C. paliurus* from sampling locations (JZS, MC, WF, MW, and SN) (mg/g, mean ± SD). JZS from Jinzhongshan, Guangxi province; MC from Muchuan, Sichuan province; WF from Wufeng, Hubei province; MW from Meiwu, Zhejiang province; SN from Suining, Hunan province. Different letters indicate significant differences (*p* < 0.05 by Duncan’s test) between locations (*n* = 3) (nd, not detected).

Compounds	LOD (ng/mL)	LOQ (ng/mL)	JZS (mg/g)	MC (mg/g)	WF (mg/g)	MW (mg/g)	SN (mg/g)
3-*O*-caffeoylquinic acid (P1)	64.25	214.21	0.35 ± 0.011b	1.38 ± 0.153a	0.39 ± 0.003b	0.45 ± 0.028b	0.31 ± 0.003b
4-*O*-caffeoylquinic acid (P2)	57.93	197.58	0.03 ± 0.001d	0.08 ± 0.003c	0.28 ± 0.003a	0.11 ± 0.003b	0.08 ± 0.002c
4,5-di-*O*-caffeoylquinic acid (P3)	58.97	201.22	0.05 ± 0.003d	0.10 ± 0.001c	0.42 ± 0.028a	0.16 ± 0.003b	0.08 ± 0.003cd
quercetin-3-*O*-glucuronide (F1)	40.28	128.74	2.15 ± 0.010b	1.57 ± 0.006d	2.11 ± 0.003c	2.38 ± 0.005a	0.78 ± 0.003e
quercetin-3-*O*-galactoside (F2)	52.94	174.17	0.47 ± 0.005c	0.24 ± 0.003d	0.52 ± 0.003b	0.57 ± 0.005a	0.20 ± 0.003e
isoquercitrin (F3)	58.42	192.52	0.22 ± 0.005b	0.32 ± 0.006a	0.18 ± 0.006c	0.21 ± 0.013b	0.08 ± 0.001d
kaempferol-3-*O*-glucuronide (F4)	43.98	153.14	1.33 ± 0.001b	0.86 ± 0.001d	1.88 ± 0.001a	0.95 ± 0.001c	0.51 ± 0.001e
kaempferol-3-*O*-glucoside (F5)	53.85	187.37	0.18 ± 0.001b	0.20 ± 0.001a	0.12 ± 0.001c	0.08 ± 0.001d	0.05 ± 0.001e
quercetin-3-*O*-rhamnoside (F6)	62.48	199.32	0.23 ± 0.001a	0.11 ± 0.001d	0.20 ± 0.001b	0.19 ± 0.001c	nd
kaempferol-3-*O*-rhamnoside (F7)	64.13	211.81	2.30 ± 0.001a	1.39 ± 0.001d	1.78 ± 0.001b	1.50 ± 0.006c	0.68 ± 0.001e
total phenolic acids (TPA)	-	-	0.43 ± 0.014c	1.57 ± 0.154a	1.09 ± 0.023b	0.71 ± 0.034c	0.47 ± 0.007c
total flavonoids (TF)	-	-	6.88 ± 0.011a	4.70 ± 0.003d	6.80 ± 0.006b	5.90 ± 0.008c	2.30 ± 0.003e
total phenolics (TPC)	-	-	7.31 ± 0.025b	6.27 ± 0.152d	7.89 ± 0.017a	6.61 ± 0.042c	2.77 ± 0.008e

**Table 3:** Pearson correlation coefficients between phenolic compounds and antioxidant index (*n* = 15). DPPH (2,2-diphenyl-1-picrylhydrazyl) (IC_50_): IC_50_ of DPPH radical-scavenging activity; ferric-reducing antioxidant power (FRAP) (EC_50_): EC_50_ of FRAP; SOD: superoxide dismutase; GSH-px: glutathione peroxidase; MDA: malonaldehyde).

Assays	DPPH (IC_50_)	FRAP (EC_50_)	SOD	GSH-px	MDA
total phenolic compounds (TPC)	−0.991 **	−0.796	0.775	0.528	−0.878 *
total phenolic acids (TPA)	−0.373	−0.732	0.010	0.249	−0.090
total flavonoids (TF)	−0.950 *	−0.655	0.814	0.493	−0.903 *
3-*O*-caffeoylquinic acid (P1)	−0.195	−0.452	−0.182	0.419	0.070
4-*O*-caffeoylquinic acid (P2)	−0.312	−0.553	0.314	−0.323	−0.256
4,5-di-*O*-caffeoylquinic acid (P3)	−0.392	−0.597	0.378	−0.266	−0.331
quercetin-3-*O*-glucuronide (F1)	−0.898 *	−0.700	0.559	0.287	−0.698
quercetin-3-*O*-galactoside (F2)	−;0.716	−0.530	0.500	−0.004	−0.584
isoquercitrin (F3)	−0.687	−0.698	0.242	0.684	−0.413
kaempferol-3-*O*-glucuronide (F4)	−0.768	−0.584	0.871	0.312	−0.855
kaempferol-3-*O*-glucoside (F5)	−0.654	−0.420	0.560	0.955 *	−0.655
quercetin-3-*O*-rhamnoside (F6)	−0.939 *	−0.639	0.735	0.467	−0.848
kaempferol-3-*O*-rhamnoside (F7)	−0.874	−0.426	0.883 *	0.716	−0.954 *

* and ** indicate correlation is significant at the 0.05 and 0.01 level, respectively.

**Table 4:** Geographical and climatic information of locations for sampling *C. paliurus*.

Sample ID	Location	Latitude (N)	Longitude (E)	Mean Annual Temperature (°C)	Altitude (m)	Mean Annual Sunshine Hours (h)	Mean Annual Precipitation (mm)
JZS	Jinzhongshan, Guangxi	24°36′36″	104°57′00″	17.1	1798	1475	1200
MC	Muchuan, Sichuan	28°58′00″	103°47′00″	12.9	1200	965.3	1533
WF	Wufeng, Hubei	30°17′00″	110°80′00″	16.7	688	1533	1893
MW	Meiwu, Zhejiang	27°46′00″	119°17′00″	16.5	678	1862	1600
SN	Suining, Hunan	26°22′00″	110°07′00″	16.7	862	1348	1320

## Supplementary Materials

The following are available online. Figure S1: the representative HPLC chromatogram of 80% ethanol extract of the *C. paliurus* leaves collected from (a) JZS and (b) mixed standards.

## References

[1] Delfanian M., Esmaeilzadeh Kenari R., Sahari M.A. (2016). Utilization of Jujube fruit (*Ziziphus mauritiana* Lam.) extracts as natural antioxidants in stability of frying oil. Int. J. Food Prop..

[2] Kahl R., Kappus H. (1993). Toxicology of the synthetic antioxidants BHA and BHT in comparison with the natural antioxidant vitamin E. Z. Lebensm. Unters. Forsch..

[3] Guo X.D., Ma Y.J., Parry J., Gao J.M., Yu L.L., Wang M. (2011). Phenolics content and antioxidant activity of tartary buckwheat from different locations. Molecules.

[4] Liu Y., Cao Y.N., Fang S.Z., Wang T.L., Fu X.X. (2018). Antidiabetic effect of *Cyclocarya paliurus* leaves depends on the contents of antihyperglycemic flavonoids and antihyperlipidemic triterpenoids. Molecules.

[5] Jia X., Luo H., Xu M., Zhai M., Guo Z., Qiao Y., Wang L. (2018). Dynamic changes in phenolics and antioxidant capacity during Pecan (*Carya illinoinensis*) kernel ripening and its phenolics profiles. Molecules.

[6] Mensor L.L., Menezes F.S., Leitão G.G., Reis A.S., Santos T.C.D., Coube C.S., Leitão S.G. (2001). Screening of Brazilian plant extracts for antioxidant activity by the use of DPPH free radical method. Phytother. Res..

[7] Čopra-Janićijević A., Čulum D., Vidic D., Tahirović A., Klepo L., Bašić N. (2018). Chemical composition and antioxidant activity of the endemic *Crataegus microphylla* Koch subsp *malyana* KI Chr. and Janjić from Bosnia. Ind. Crops Prod..

[8] Xie J.H., Dong C.J., Nie S.P., Li F., Wang Z.J., Shen M.Y., Xie M.Y. (2015). Extraction, chemical composition and antioxidant activity of flavonoids from *Cyclocarya paliurus* (Batal.) Iljinskaja leaves. Food Chem..

[9] Fang S., Wang J., Wei Z., Zhu Z. (2006). Methods to break seed dormancy in *Cyclocarya paliurus* (Batal) Iljinskaja. Sci. Hortic..

[10] Kurihara H., Asami S., Shibata H., Fukami H., Tanaka T. (2003). Hypolipemic effect of *Cyclocarya paliurus* (Batal) Iljinskaja in lipid-loaded mice. Biol. Pharm. Bull..

[11] Fang S., Yang W., Chu X., Shang X., She C., Fu X. (2011). Provenance and temporal variations in selected flavonoids in leaves of *Cyclocarya paliurus*. Food Chem..

[12] Deng B., Shang X., Fang S., Li Q., Fu X., Su J. (2012). Integrated effects of light intensity and fertilization on growth and flavonoid accumulation in *Cyclocarya paliurus*. J. Agric. Food Chem..

[13] Cao Y., Fang S., Yin Z., Fu X., Shang X., Yang W., Yang H. (2017). Chemical Fingerprint and Multicomponent Quantitative Analysis for the Quality Evaluation of *Cyclocarya paliurus* Leaves by HPLC-Q-TOF-MS. Molecules.

[14] Jia W., Gao W., Tang L. (2003). Antidiabetic herbal drugs officially approved in China. Phytother. Res..

[15] Ahmad Aufa Z., Hassan F.A., Ismail A., Mohd Yusof B.N., Hamid M. (2014). Chemical compositions and antioxidative and antidiabetic properties of underutilized vegetable palm hearts from *Plectocomiopsis geminiflora* and *Eugeissona insignis*. J. Agric. Food Chem..

[16] Altiparmak I.H., Erkus M.E., Gunebakmaz O. (2016). Oxidative stress is associated with not only coronary artery disease on statin therapy but also diabetes mellitus and hypertension. Indian Heart J..

[17] Bandeira S.D.M., Guedes G.D.S., Fonseca L.J.S.D., Pires A.S., Gelain D.P., Moreira J.C.F., Goulart M.O.F. (2012). Characterization of blood oxidative stress in type 2 diabetes mellitus patients: Increase in lipid peroxidation and SOD activity. Oxid. Med. Cell. Longev..

[18] Yang H., Jin X., Lam C.W.K., Yan S.K. (2011). Oxidative stress and diabetes mellitus. Clin. Chem. Lab. Med..

[19] Kowald A., Hamann A., Zintel S., Ullrich S., Klipp E., Osiewacz H.D. (2012). A systems biological analysis links ROS metabolism to mitochondrial protein quality control. Mech. Ageing Dev..

[20] Chen H., Yu M., Li M., Zhao R., Zhu Q., Zhou W., Zhao W. (2012). Polymorphic variations in manganese superoxide dismutase (MnSOD), glutathione peroxidase-1 (GPX1), and catalase (CAT) contribute to elevated plasma triglyceride levels in Chinese patients with type 2 diabetes or diabetic cardiovascular disease. Mol. Cell. Biochem..

[21] Xi W., Zhang Y., Sun Y., Shen Y., Ye X., Zhou Z. (2014). Phenolic composition of Chinese wild mandarin (*Citrus reticulata* Balnco.) pulps and their antioxidant properties. Ind. Crops Prod..

[22] Liu Y., Qian C., Ding S., Shang X., Yang W., Fang S. (2016). Effect of light regime and provenance on leaf characteristics, growth and flavonoid accumulation in *Cyclocarya paliurus* (Batal) Iljinskaja coppices. Bot. Stud..

[23] Bessada S.M., Barreira J.C., Barros L., Ferreira I.C., Oliveira M.B.P. (2016). Phenolic profile and antioxidant activity of *Coleostephus myconis* (L.) Rchb. f.: An underexploited and highly disseminated species. Ind. Crops Prod..

[24] Li X.C., Fu X.X., Shang X.L., Yang W.X., Fang S.Z. (2017). Natural population structure and genetic differentiation for heterodicogamous plant: *Cyclocarya paliurus* (Batal.) Iljinskaja (Juglandaceae). Tree Genet. Genomes.

[25] Sosa T., Alías J.C., Escudero J.C., Chaves N. (2005). Interpopulational variation in the flavonoid composition of *Cistus ladanifer* L. exudate. Biochem. Syst. Ecol..

[26] Contreras-Calderón J., Calderón-Jaimes L., Guerra-Hernández E., García-Villanova B. (2011). Antioxidant capacity, phenolic content and vitamin C in pulp, peel and seed from 24 exotic fruits from Colombia. Food Res. Int..

[27] Fatiha B., Didier H., Naima G., Khodir M., Martin K., Léocadie K., Pierre D. (2015). Phenolic composition, in vitro antioxidant effects and tyrosinase inhibitory activity of three Algerian *Mentha* species: *M. spicata* (L.), *M. pulegium* (L.) and *M. rotundifolia* (L.) Huds (Lamiaceae). Ind. Crops Prod..

[28] Huang W., Xue A., Niu H., Jia Z., Wang J. (2009). Optimised ultrasonic-assisted extraction of flavonoids from *Folium eucommiae* and evaluation of antioxidant activity in multi-test systems in vitro. Food Chem..

[29] Wang L., Yang X., Qin P., Shan F., Ren G. (2013). Flavonoid composition, antibacterial and antioxidant properties of tartary buckwheat bran extract. Ind. Crops Prod..

[30] Tohidi B., Rahimmalek M., Arzani A. (2017). Essential oil composition, total phenolic, flavonoid contents, and antioxidant activity of *Thymus* species collected from different regions of Iran. Food Chem..

[31] He J., Huang B., Ban X., Tian J., Zhu L., Wang Y. (2012). In vitro and in vivo antioxidant activity of the ethanolic extract from *Meconopsis quintuplinervia*. J. Ethnopharmacol..

[32] Lee J.S. (2006). Effects of soy protein and genistein on blood glucose, antioxidant enzyme activities, and lipid profile in streptozotocin-induced diabetic rats. Life Sci..

[33] Boots A.W., Haenen G.R., Bast A. (2008). Health effects of quercetin: From antioxidant to nutraceutical. Eur. J. Pharmacol..

[34] Calderon-Montano J.M., Burgos-Morón E., Pérez-Guerrero C., López-Lázaro M. (2011). A review on the dietary flavonoid kaempferol. Mini-Rev. Med. Chem..

[35] Xiao J., Muzashvili T.S., Georgiev M.I. (2014). Advances in the biotechnological glycosylation of valuable flavonoids. Biotechnol. Adv..

[36] Liu Y., Fang S.Z., Zhou M.M., Shang X.L., Yang W.X., Fu X.X. (2018). Geographic variation in water-soluble polysaccharide content and antioxidant activities of *Cyclocarya paliurus* leaves. Ind. Crops Prod..

[37] Brand-Williams W., Cuvelier M.E., Berset C. (1995). Use of a free radical method to evaluate antioxidant activity. LWT-Food Sci. Technol..

[38] Oyaizu M. (1986). Antioxidative activity of browning products of glucosamine fractionated by organic solvent and thin layer chromatography. Nippon Shokuhin Kogyo Gakkaishi.

[39] Wang Q., Jiang C., Fang S., Wang J., Ji Y., Shang X., Zhang J. (2013). Antihyperglycemic, antihyperlipidemic and antioxidant effects of ethanol and aqueous extracts of *Cyclocarya paliurus* leaves in type 2 diabetic rats. J. Ethnopharmacol..

