# Supplementary material for: Geographic Variation in the Chemical Composition and Antioxidant Properties of Phenolic Compounds from Cyclocarya paliurus (Batal) Iljinskaja Leaves
Source: Molecules. 2018 Sep 24;23(10):2440. doi: 10.3390/molecules23102440 (PMC6222593; doi:10.3390/molecules23102440)

Figure S1. The representative HPLC chromatogram of 80% ethanol extract of the *C. paliurus* leaves collected from JZS (a), and mixed standards (b). 1. 3-*O*-caffeoylquinic acid; 2. 4-*O*-caffeoylquinic acid; 3. quercetin-3-*O*-glucuronide; 4. quercetin-3-*O*-galactoside; 5. isoquercitrin; 6. kaempferol-3-*O*-glucuronide; 7. kaempferol-3-*O*-glucoside; 8. quercetin-3-*O*-rhamnoside; 9. 4,5-di-*O*-caffeoylquinic acid; 10. kaempferol-3-*O*-rhamnoside.

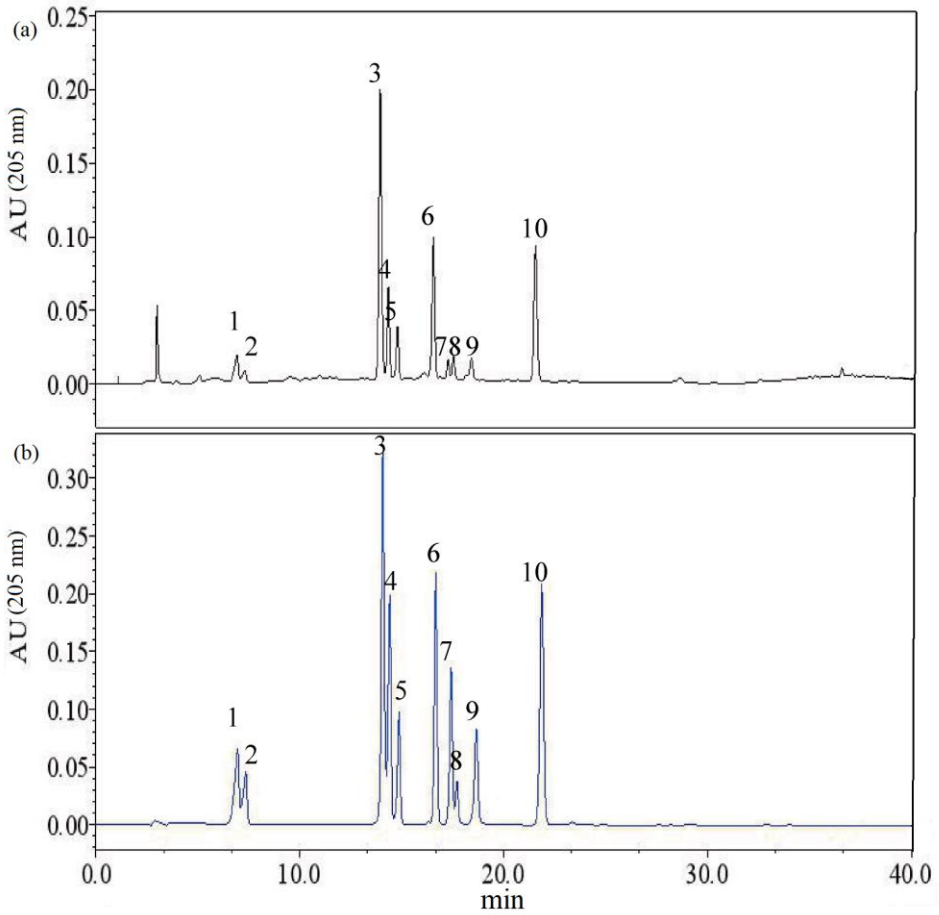

Supplement: Supplementary file 1 [file molecules-23-02440-s001.pdf]
